# *Var *transcription profiling of *Plasmodium falciparum *3D7: assignment of cytoadherent phenotypes to dominant transcripts

**DOI:** 10.1186/1475-2875-7-14

**Published:** 2008-01-14

**Authors:** Uta Gölnitz, Letusa Albrecht, Gerhard Wunderlich

**Affiliations:** 1 Department of Parasitology, Institute of Biomedical Sciences, University of São Paulo, Avenida Prof. Lineu Prestes 1374, São Paulo – SP, Brazil

## Abstract

**Background:**

Cytoadherence of *Plasmodium falciparum*-infected red blood cells is mediated by *var *gene-encoded *P. falciparum *erythrocyte membrane protein-1 and host receptor preference depends in most cases on which of the 50–60 *var *genes per genome is expressed. Enrichment of phenotypically homogenous parasites by panning on receptor expressing cells is fundamental for the identification of the corresponding *var *transcript.

**Methods:**

*P. falciparum *3D7 parasites were panned on several transfected CHO-cell lines and their *var *transcripts analysed by i) reverse transcription/PCR/cloning/sequencing using a universal DBLα specific oligonucleotide pair and ii) by reverse transcription followed by quantitative PCR using 57 different oligonucleotide pairs.

**Results:**

Each cytoadherence selected parasite line also adhered to untransfected CHO-745 cells and upregulation of the *var *gene PFD995/PFD1000c was consistently associated with cytoadherence to all but one CHO cell line. In addition, parasites panned on different CHO cell lines revealed candidate *var *genes which reproducibly associated to the respective cytoadherent phenotype. The transcription profile obtained by RT-PCR/cloning/sequencing differed significantly from that of RT-quantitative PCR.

**Conclusion:**

Transfected CHO cell lines are of limited use for the creation of monophenotypic cytoadherent parasite lines. Nevertheless, 3D7 parasites can be reproducibly selected for the transcription of different determined *var *genes without genetic manipulation. Most importantly, *var *transcription analysis by RT-PCR/cloning/sequencing may lead to erroneous interpretation of *var *transcription profiles.

## Background

*Plasmodium falciparum *the causative agent of tropical malaria still kills over one million people per year, mostly children under five years in sub-Saharan Africa [1]. One major virulence factor of this parasite is the highly variant *Plasmodium falciparum *erythrocyte membrane protein-1 (PfEMP-1) family [2]. Members of this family appear at the surface infected red blood cell, during the trophozoite stage. Their large, highly variable ectodomains, consist of several subdomains (termed Duffy binding-like, DBL, and cystein rich interdomain regions, CIDR), which interact with a number of cellular host receptors such as CD36, ICAM-1, CSA, PECAM, E-Selectin, VCAM and others (reviewed by [3]). This interaction causes the retention of mature blood stage forms in the tissue which express the cited receptors and is believed to result in pathogenic processes associated with malaria [4]. Each infected red blood cell (IRBC) displays only one PfEMP-1 allele at its surface and this selective expression is controlled at the transcriptional level [5] by allelic exclusion [6,7]. It is believed that only one of the approximately fifty *var *promoters is active and concomitantly localized to a specific compartment at the nuclear periphery [8-10]. The transcriptional activity of *var *promoters is apparently determined by histone modifications [11-14], which may then permit or inhibit the recruitment of yet largely unknown transcription factors. The pattern of *var *transcription is inherited through several generations [15,16] despite the absence of DNA methylation [17] and switches in the transcription result in altered antigenic phenotypes.

Several groups tried to correlate disease outcomes with adhesive IRBC phenotypes, with sometimes contradicting outcomes for ICAM-1 and/or CD36-mediated adhesion [18,19]. The excessive CSA-mediated adhesion of IRBC was clearly correlated to severe malaria outcomes in primigravids [20] and the PfEMP1_csa_-encoding var2csa gene, upregulated in CSA-adhesive parasites, was analysed in detail [21] and the structure of its crucial DBLγ domain mapped [22].

So far, few studies associated the *var *genes to adhesive phenotypes in cultivated field isolates. One Indian isolate was obtained after enrichment of an ICAM-1-adhesive phenotype [23]. Parasites obtained from the placenta of primigravid women frequently transcribe var2csa and adhere to CSA found in abundance on syncithiotrophoblasts [24]. The primary *var *transcript sequence can be of importance for the design of specific PfEMP-1 domains that may be used as a vaccine inducing antibodies, which then inhibit or decrease cytoadherence of IRBCs, as shown for CD36-binding CIDR [25] and CSA-binding DBLγ domains [26]. Also, for the elucidation of the epigenetic control mechanisms that orchestrate *var *gene transcription it may be of interest to reproducibly obtain parasites with a defined *var *locus switched on or off in its native, unmodified genomic context. The enrichment of adhesive phenotypes is achieved by the panning procedure, where cytoadherent IRBCs are incubated either on isolated receptor molecules [15] or on receptor-expressing cells, such as amelanotic melanoma cells expressing CD36 [27], Saimiri brain endothelial cells (SBEC) expressing CSA and/or CD36 and ICAM-1 [28], human lung endothelial cells (HLEC) expressing CD36 and ICAM-1 [29], human choriocarcinoma cells expressing CSA and CD36 [30] or Chinese hamster ovary cells (CHO) transfected with the respective receptors [31]. After several rounds of panning and amplification of the parasites a sufficiently high homogeneity of the cytoadherent phenotype is obtained which then permits functional or transcript analysis. The CHO cell lineages are especially convenient since they express the receptors in a constitutive fashion without the need of external stimulation as it is the case for SBEC or HLEC. However, the CHO cell lineage has the disadvantage that there is a – to date unknown – receptor which is recognized by equally unknown parasite encoded PfEMP1 or other molecules which may mask the desired receptor-ligand interaction [32]. In order to estimate the viability of the transfected CHO cell lines in the enrichment of relevant adhesive phenotypes and later transcript analysis, *var *gene transcription was monitored of different *P. falciparum *3D7 parasite lines panned on receptors expressed on stably transfected CHO-cell lines [31,33]. *Var *transcripts were then analysed by reverse transcription followed by quantitative real time PCR (RT-qPCR). In addition, we also evaluated if the frequently used approach of reverse transcription followed by PCR, fragment cloning and sequence read counting (RT-PCRcsc), is reliable to estimate dominant *var *transcripts in *P. falciparum*. This issue is of major importance since field isolates can currently be tested for their *var *transcripts only by this approach.

## Methods

### Parasites and cell lines

*Plasmodium falciparum *3D7 parasites [34], a cloned line derived from isolate NF54, were cultured and sorbitol-synchronized according to previously published methods [35,36]. Stable transfectants of CHO expressing CSA (CHO-K1), CD36, E-Selectin, ICAM-1, VCAM [31,33] and the CSA negative CHO variant *pgsA *(CHO-745, [37,38], a gift from Artur Scherf, Institut Pasteur, Paris) were cultured in RPMI 1640 containing 10% FCS, 40 mg/L gentamycin in a 5% CO_2 _atmosphere at 37°C. All cultures were tested mycoplasma-negative by PCR.

### Panning of *P. falciparum *3D7 parasites on CHO cells

Selection of IRBC for stationary adhesion to each of the different CHO cells was carried out basically as previously described [6]. Briefly, trophozoite-stage (26–28 hpi) infected erythrocytes were enriched by Plasmagel^® ^flotation and resuspended in RPMI 1640 pH 6.8 containing 10% pooled heat-inactivated human plasma. 10^7^-10^8 ^IRBC were then incubated with confluent CHO cell monolayers grown in 25 cm^2 ^culture flasks for 1 h with gentle agitation every 15 min. Non-adherent infected erythrocytes were washed away three times in RPMI 1640, pH 6.8 by direct aspiration. Bound infected erythrocytes were detached from cells by washing with RPMI 1640, pH 7,4 containing 10% plasma and returned to culture, adjusting the hematocrit to 5%. Cultures were grown to 2–6% parasitaemia before repeating this process. Pannings were continued until no more increase in adherence was observed (at least 5 times), and the phenotype was maintained by panning every 2–3 weeks. For the quantification of cytoadherence, 5*10^6 ^infected red blood cells were panned as above over 90% confluent CHO-cells (two days after trypsinization) cultivated in 3 cm diameter culture plates.

### RNA isolation, cDNA production and real-time PCR

Total RNA was isolated from parasites at most two reinvasion cycles after the last panning procedure and the Trizol LS Reagent (Invitrogen) was used as previously described [39]. RNA was then treated with Deoxyribonuclease I (Fermentas) to degrade contaminating genomic DNA. cDNA synthesis was performed with Mu-MLV Reverse Transcriptase (Fermentas) as described by the manufacturer using random hexamer primers (Bioneer). For each cDNA synthesis reaction, a control reaction without reverse transcriptase was done with identical amounts of template.

Real-time qPCRs were carried out using the Realplex 2/2 thermocycler (Eppendorf, Hamburg, Germany). Reactions were performed in 15 μl volumes using Biotools QuantiMix EASY SYG KIT (Biotools, B&M Labs, S.A.) and 0,3 mM of each primer. In order to measure transcription from all *var *loci present in the 3D7 genome, we employed the previously described primer set [21]. The qPCR conditions were 95°C for 2 min followed by 40 cycles of 95°C for 30 s, 54°C for 40 s, and 68°C for 50 s. Specificity of amplification was ascertained by melting-curve analysis of each PCR product. All runs were done in triplicate and yielded virtually identical Ct (cycle threshold) values. The ΔCt for each individual primer pair was determined by subtracting the measured Ct value from the Ct value of the control seryl-tRNA synthetase (PF07_0073)[40]. Relative copy numbers (RCN) were then obtained with the formula: RCN = 2^-ΔCt^. Two independent RNAs were tested for each receptor-ligand phenotype. The Ct values of the internal control were always below 30 cycles.

### Cloning and sequencing of DBL1α region

cDNAs were amplified with the universal DBL1α primer pair [41], purified from agarose gels and cloned using the InsTA-vector cloning system (Fermentas). For the cDNA from 3D7-Selectin and 3D7-ICAM-1 selected parasites, 32 inserts were sequenced and identified by alignment with fragments of 3D7 from the PlasmoDB website.

## Results

### Panning of 3D7 parasites creates highly adhesive parasites which cross-adhere to CHO-745 cells

After five rounds of panning on each of the CHO-cell lines the adhesive phenotype remained stable and no further increase in the ratio adhered parasites per CHO-cell was observed. Upon saturating conditions (excess of IRBC over CHO cells) the adhesive IRBC phenotypes were highly similar for each cell line and the cytoadherence expressed as IRBC/100 cells was 300–800 and did not differ consistently between phenotypes. Interestingly, when each CHO-receptor-panned parasite line was analyzed for cytoadherence in CHO-745 cells, all different phenotypes also adhered to these cells, although to a lower amount (100–300 IRBC/100 cells). Upon quantification using fewer parasites than in the cytoadherence selection process, differences in the quantity of adhered parasites were observed (Table 1). 3D7-745, 3D7-ICAM and the 3D7-VCAM parasites showed the highest adherence values.

**Table 1:** Cytoadherence of differently selected 3D7 parasites selected for adhesion on the indicated CHO-cell lines

	CHO-745	CHO-K1	CHO-CD36	CHO-ICAM	CHO-Sel	CHO-VCAM
3D7	7 ± 2	0	12 ± 4	7 ± 3	5 ± 1	4 ± 2
3D7-745	142 ± 13	120 ± 23	15 ± 3	253 ± 22	28 ± 5	270 ± 26
3D7-K1	25 ± 3	29 ± 5	32 ± 8	45 ± 8	45 ± 3	34 ± 4
3D7-CD36*	16 ± 3	3 ± 1	62 ± 7	33 ± 5	25 ± 5	23 ± 6
3D7-ICAM*	40 ± 9	40 ± 7	152 ± 12	235 ± 18	141 ± 13	151 ± 17
3D7-Selectin	13 ± 2	16 ± 3	21 ± 5	50 ± 8	34 ± 3	15 ± 5
3D7-VCAM	51 ± 11	51 ± 8	92 ± 12	226 ± 21	151 ± 14	51 ± 5

A stock of freshly panned parasites was thawed, put into culture and after 4–6 reinvasions, 5*10^6 ^IRBC (at trophozoite stage) were incubated on the indicated CHO-cell lines, washed and counted. For each cell line, 1000 cells and the adhering parasites were counted and cytoadherence was expressed as IRBC per 100 cells. Phenotypes which significantly (Chi-square method, *p *< 0,05) adhered more to the CHO cell line for which they were selected than to any other cell line are marked with an asterisk.

### Transcription analysis of selected IRBC indicates a *var *gene associated with cytoadherence to the unknown receptor on CHO-745 cells

**Figure 1:**
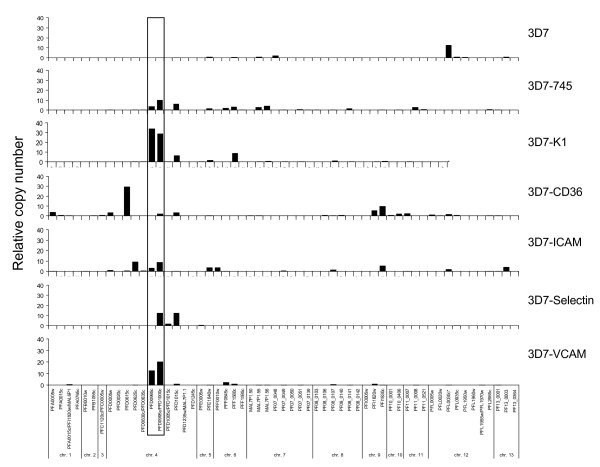
***var *transcription in different adhesive 3D7 phenotypes**. Transcription levels were measured by real-time PCR using primers specific for each of the 57 different *var *genes identified in the *P. falciparum *3D7 genome. All values are presented as relative copy numbers, calibrated to the housekeeping gene *seryl-tRNA synthetase *(PF07_0073). Top panel: Transcription pattern of 3D7 wildtype parasites demonstrated low *var *gene expression in general. Second panel: 3D7 parasites selected on CHO-475 cells demonstrated expression of *var *genes located on chromosome 4 – PFD0995c/PFD1000c, similar to 3D7 parasites selected on CHO-K1 cells (CHO-CSA, Third panel). Fourth panel: 3D7-CD36 demonstrated expression of the PFD0615c *var *gene located on chromosome 4, but not the CHO-745 related *var *genes PFD0995c/PFD1000c. Fifth panel: 3D7-ICAM1 cells transcribed PFD0625c but also PFD0995c/PFD1000c. Sixth panel: 3D7 parasites selected on CHO-Selectin cells showed expression of PFD1000c and PFD1015c on chromosome 4. Seventh panel: 3D7-VCAM parasites transcribed PFD0995c/PFD1000c and did not show any other upregulated *var *gene.

In the following experiment, parasites selected as above were harvested in young trophozoite stage and their *var *transcripts analyzed by RT-qPCR (Figure 1). The unselected parasites transcribed mainly the *var *gene PFL0030c (var2csa), however, only weak adherence of these parasites to CHO-K1 cells was observed (<10 IRBC/100 cells), indicating that cytoadherence to CSA is weak or absent in the 3D7 parasite line. Repeated selection on the CSA-knockout CHO-745 cells resulted in increased transcription of PFD995c/PFD1000c *var *genes, which share similar promoters and approximately 2831 nt in the 5'portion of each gene [42,43]. Other *var *genes from chromosomes 6, 7 and 11 were also found in higher quantities. Notably, upon selection on CHO-K1 (unmodified, CSA expressing cell line), the same dominant transcript PFD0995c was obtained and transcripts from *var *locus PFF1580c on chromosome 6 were also detected.

When parasites were selected on CHO-CD36, no significant amounts of the PFD0995/1000c transcripts were observed, instead, transcription from the PFD0615c locus was activated. Also, minor quantities of *var *transcripts from chromosome 9 were observed. Interestingly, this parasite line also bound well to untransfected CHO-745 cells (Table 1).

The selection of 3D7 on CHO-ICAM-1 demonstrated the transcription of principally PFD995c/1000c and PFD0625c. A number of other *var *loci were also found active, principally on chromosomes 5, 6, 9 and 13.

The selection of cytoadherent parasites on CHO-Selectin cells yielded a parasite line expressing two main *var *transcripts PFD1000c and PFD1015c while all other *var *loci seemed silenced. In the last experiment, the transcripts of CHO-VCAM selected 3D7 parasites were monitored. As shown in Figure 1, *var *transcription occurred almost exclusively from the *var *locus PFD995c/1000c. Since the primer pair for PFD0995c/PFD1000c co-amplifies PFD0995c and PFD1000c, and the PFD0995c transcript alone was found in higher quantities, most transcripts seem to originate from the PFD0995c locus, similar to 3D7-K1 but in contrast to 3D7-745 parasites, which show more transcripts from the PFD1000 locus.

**Figure 2:**
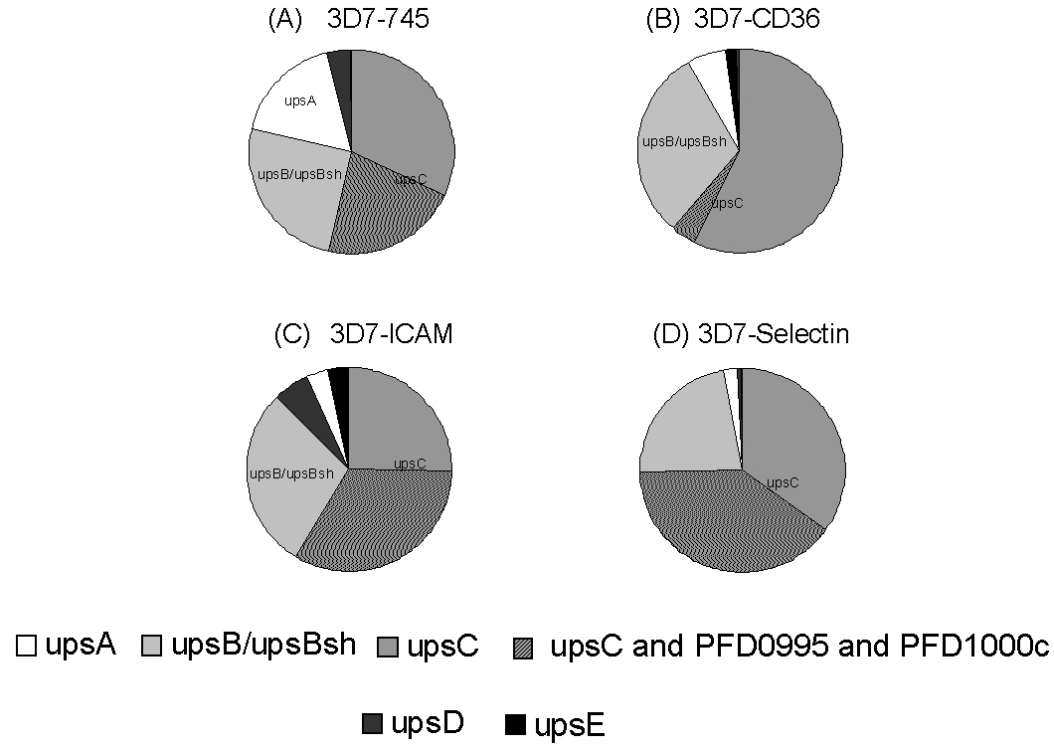
***var *promoter profile of different adhesive parasites phenotypes**. The pie charts present relative levels of transcription of each *var *promoter type from parasites selected on – upsA, upsB/upsBsh, upsC, upsD and upsE. In hatched dark grey, the fraction of transcripts from upsC *var *genes PFD0995/PFD1000c are highlighted.

The herein used stationary assay of cytoadherence is believed to select for the strongest binding parasite phenotype. In order to elucidate from which promoter type the most abundant transcripts were generated, the relative *var *transcript copy numbers of each transcript were grouped corresponding to their promoter type upsA, B, C, D and E. As shown in Figure 2, most of the transcribed *var *genes were from upsC promoters, even when omitting the signal from the PFD0995/PFD1000c locus.

### Significant differences of detected *var *transcripts by RT-PCRcsc versus RT-qPCR analysis

*Var *gene transcription in field isolates was frequently analyzed by RT-PCR analysis followed by cloning and semi-quantitative clone analysis [44-47] and due to the high *var *gene variability this is the only method to monitor *var *gene transcription in field isolates. In the following, an evaluation was done whether *var *transcription measured by RT-PCRcsc matched the results obtained by RT-qPCR. Two adhesive phenotypes were selected for this analysis: The CHO-Selectin phenotype which shows the transcription of the *var *genes PFD1000 and PFD1015c and the CHO-ICAM-1 phenotype which shows the CHO-745 associated PFD995/1000c transcript plus the possibly ICAM-1-binding related PFD0625c transcript.

**Figure 3:**
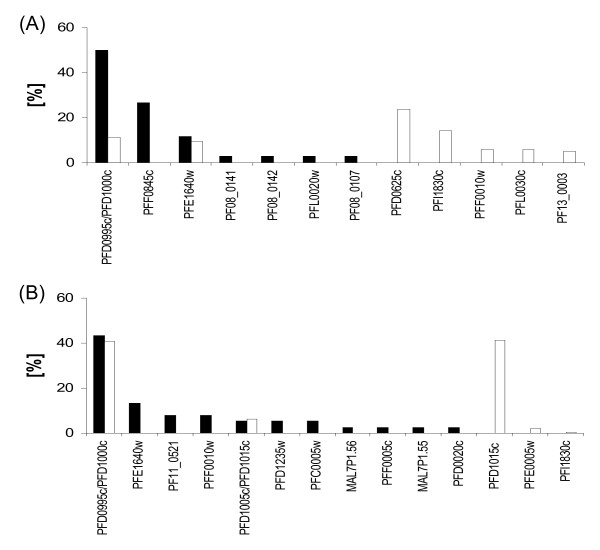
**A comparison of RT-PCRcsc and RT-qPCR methods**. 32 clones of each RT-PCRcsc experiment were sequenced and the sequence reads plotted against the sequence ID of each tag (black bars). In white bars the results for RT-qPCR are shown. (A) 3D7-ICAM-1 (B) 3D7-Selectin.

After RT-PCR and cloning of fragments generated on RNAs also used in the RT-qPCR experiment, the dominant transcripts were compared, deduced from the number of sequence reads, with the qPCR results. Surprisingly, the dominant transcript in CHO-Selectin selected parasites was exclusively PFD0995c/PFD1000c and not a single clone carrying the PFD1015c transcript was observed by this method (Figure 3). When RNA from the CHO-ICAM-1 selected parasites was analysed, the mainly observed transcript was PFD0995c/PFD1000c and the second abundant transcript was PFF0845c. The var1csa transcript (PFE1640w, truncated in 3D7 parasites), was also detected. However, the transcript PFD0625c was not observed (Figure 3). Taken together, these data indicate that in the conditions applied herein, RT-PCR followed by fragment cloning and sequence read analysis leads to results different from those obtained by RT-qPCR.

## Discussion

In the present study, a practical and simple system for the analysis and selection of *P. falciparum *adhesive phenotypes and their *var *transcripts was evaluated. This system was so far used only for the diagnosis of cytoadherent *P. falciparum *isolates [31,44], but not for the deliberate selection and enrichment of adhesive phenotypes for posterior ligand-receptor interaction or other analyses. Most of the herein used receptors are also commercially available as purified molecules, however, either these molecules are so expensive that they turn the use in quantitative selection experiments unviable or they are not displayed properly for recognition upon coating of supports such as Petri dishes or culture flasks. Also, in the case of ICAM-1 and CD36, free ICAM-1 or CD36 molecules do not detach IRBC interacting with these molecules on the cell surface, although interaction may be abrogated by specific antibodies against ICAM-1 or CD36 [48]. Therefore, the selection of parasites by specific detachment of adhered parasites, as it is feasible for the interaction of IRBC with CSA expressed on *Saimiri *brain endothelial cells [49], proved impossible.

A major handicap of the herein used system is the yet unknown receptor present on CHO-745 cells which is readily recognized by 3D7 parasites as previously described [32]. These authors demonstrated a phenotype which could be inhibited by anti-CD36 monoclonal antibody, indicating that CHO-745 may carry a CD36-like receptor on its surface. They also showed that protein A pretreated parasites were significantly cytoadherence-inhibited (although adhesion was not totally abrogated) indicating also an interference with opsonizing IgM antibodies. Since the binding ligand of 3D7 IRBC selected on CHO-745 was trypsin sensitive, the hypothesis was that the interaction IRBC to CHO-745 may at least partly be caused by PfEMP1 and anticipated the detection of specific *var *transcripts, which encode the PfEMP1 responsible for this unknown receptor. Considering the existence of this unknown receptor and supposedly uniform transgene-receptor expression on CHO-CD36, CHO-VCAM, CHO-ICAM-1, CHO-Selectin and CHO-K1 (not specifically tested for herein), the model will only be able to select for strong binding ligands, since IRBC will otherwise be selected for the unknown receptor. Its appearance in all but one selected 3D7 parasite line suggests the possibility that the unknown receptor is recognized by PfEMP1 encoded by PFD0995c/PFD1000c.

Upon analysis of the transcription profile of 3D7 selected on CSA-expressing CHO-K1 cells, no specific increased transcript abundance of var2csa was observed. In contrast to the FCR3 strain, the 3D7 parasite line was only poorly selectable on purified fixed CSA (data not shown and [21]). In a panning experiment using purified CSA, the 3D7 strain also showed decreased affinity for this receptor in comparison to other strains [50]. This possibly explains why no var2csa upregulation was observed, and the *var *transcription profile was somehow similar to the CHO-745-panned parasites. Other authors obtained the var2csa transcribing phenotype by the selection of NF54 which is isogenic with 3D7 [21,51]. A similar effect was observed for the CHO-VCAM phenotype, although it is unknown if 3D7 encodes a competent ligand for this molecule. To date, no *var *gene in any strain was associated to VCAM-adhesion, although this receptor seems recognizable by PfEMP1 [52]. Strong ligands with high binding affinities are expected to override adhesion to the unknown receptor. In the case of 3D7, CD36 seems to be readily recognized and the CHO-CD36 adherent parasites contained very low levels of the PFD0995c/PFD1000c transcript. On the other hand, 3D7-CD36 parasites also adhered to CHO-745 cells, as seen already by other groups [32], without transcribing PFD0995c/PFD1000c. The most abundant *var *transcript in this parasite line was PFD0615c. Although never functionally examined, this gene contains a CIDR domain which is grouping with other CIDR domains that are in fact CD36 binder [43]. This indicates that PFD0615c encodes a stronger binding CIDR domain than PFD0995c/PFD1000c, considered as a moderate CIDR-binder [43]. The cytoadherence of 3D7-CD36 to each other cell line including CHO-745 may be explained by the assumption that the recognized although unknown receptor on CHO-745 is CD36-like as proposed by Andrews and colleagues [32].

E-Selectin was detected as a receptor for cytoadherence [52], yet, no isolate or strain was specifically selected for this specific phenotype. Also, cytoadherence to this receptor seems to be a rare event [53] and was never clearly identified as the cause of severe malaria, although E-Selectin in its soluble form is increased in acute phase malaria [54]. It is also unknown which PfEMP1-domain mediates cytoadherence to E-Selectin. In our studies, the *var *transcript PFD1015 was clearly associated to CHO-E-Selectin adherence. In addition the *var *gene PFD1000c representing the supposed ligand for the unknown receptor of CHO-745 cells was also present, explaining the cross-adherence to CHO-745 and other CHO cells. The cytoadherence to CHO-ICAM-1 showed that transcript PFD0625c was found in elevated levels. Since ICAM-1, as well as CD36, is a relative abundant receptor in man, a number of competent PfEMP1 molecules are expected to be present in the 3D7 parasite line. Indeed, a couple o *var *genes were found transcribed together with the CHO-745 specific transcripts PFD0995c/PFD1000c. The *var *gene PFD0625c, however, contains no DBLβ/C2 domain important for ICAM-1-binding [55,56], indicating that this *var *gene may not encode the ICAM-1-recognizing PfEMP1. Interestingly, other authors found a comparable result when analysing *var *transcripts by microarray analysis. Upon multiple pannings on purified ICAM-1 the most transcribed *var *gene detected did not contain a DBL-β/C2 domain [50]. In another study, still another *var *gene than PFD0625c was found in 3D7 parasites selected for ICAM-1 adhesion [57]. This indicates that it is not clear whether the CHO-ICAM-1 adhesive phenotype really binds to human ICAM-1.

The relative transcript quantities against their corresponding upstream regions were then clustered [58]. Most of the transcripts in selected parasites were from upsC and upsB promoters and only in minor quantities transcripts from subtelomeric locations were observed, even when discarding the dominantly found PFD0995c/1000c transcript. It is possible that strong binding PfEMP1-coding *var *genes are found in centromeric localizations, while weaker binding PfEMP1 molecules are encoded by subtelomeric *var *genes, prone to ectopic recombination and rearrangements [59]. Clearly, the unambiguous identification of ligands and receptors is necessary and may confirm or refute this hypothesis.

The analysis of transcripts by the to-date unique applicable system of transcript analysis in field isolates showed a significant discordance to the results obtained in RT-qPCR. Of the two main transcripts that were highly represented in the CHO-Selectin binding parasites, only the PFD1000c transcript was detected by clone analysis. A similar result was shown before using even another primer pair binding upstream of the universal *var *oligonucleotides [47]. In a similarly designed study, Gatton and coworkers also postulated that the PFD1015c DBLα fragment may not be amplified by the universal *var *oligos [60]. It is puzzling why the PFD1015 DBL1α tag was not even cloned once, since it has perfect target site for the universal oligonucleotide pair used herein. In more than 500 sequence reads from diverse 3D7 cDNAs, the PFD1015 *var *DBL1α tag was encountered only once. Notably, in other studies amplifying either genomic or transcribed targets [41,57,61] the PFD1015c tag was also never observed. The discrepancy of the two approaches was also observed in other adhesive phenotypes, however, the cDNAs used in these experiments were not from the same lot as the ones tested herein by qRT-PCR (Table 2). Assuming that RT-qPCR using the described primer set is the most reliable experimental procedure to study *var *gene transcription in 3D7, the RT-PCR/cloning approach is highly misleading and not reflecting true transcript quantities, at least in the conditions used herein. This implies that data from field samples analysed by this method may also not display the true transcript levels in the corresponding parasites. There is no simple explanation for these differences between the two methods. Either, certain quantities of 3'-truncated transcripts are present, which can be reverse transcribed and amplified by DBL1α specific primers. However, two groups recently demonstrated that there is no "relaxed" transcription in ring stage parasites [5,62] and thus no significant accumulation of 3'-truncated transcripts. On the other hand, it may be speculated that an existing primer bias is exacerbated upon amplification of cDNA due to secondary structures interfering with the processivity of the Taq polymerase. Taken together, our data suggest caution when trying to correlate *var *transcripts detected by RT-PCRcsc from field isolates to clinical outcomes. The selection of parasites on transfected CHO-cells revealed reproducibly different patterns of *var *transcription in dependence on which CHO cell line was used which may be used in further analyses of receptor-ligand studies or, for example, for the elucidation of transcriptional activation or silencing of *var *loci without the need for transfection of artificial *var *promoters.

**Table 2:** Sequence read distribution in cDNA produced from 3D7 parasites panned on CHO-745, CHO-K1, CHO-CD36 and CHO-VCAM.

***var *transcript from locus**	**3D7_745**	**3D7_CSA**	**3D7_CD36**	**3D7_VCAM**
PF07_0051			1/29	1/17
PF08_0107	3/30			
PF10_001			1/29	
PFD0615c	16/30	7/16	25/29	1/17
PFD0635c	1/30			
PFD0995c				3/17
PFD1000c	3/30	1/16	1/29	
PFD1015c		1/16		
PFF0845C	6/30	7/16		1/17
PFF1580c			1/29	
PFL0020w	1/30			1/17
PFL1970w				10/17

A varying number of clones stemming from the ligation of a RT-PCR fragment of the indicated parasite lines was analysed as described. The resulting sequences are indicated on the left.

## Conclusion

Cytoadherence selection of *P. falciparum *parasites using CHO cells is significantly hindered by the existence of a competent receptor on the CHO cell lineage. This suggests that the interaction of *P. falciparum *3D7 IRBC to this perhaps CD36-like receptor is mediated by the PfD0995c/1000c encoded PfEMP1. Comparative analysis of RT-PCRcsc and RT-qPCR revealed that RT-PCRcsc is misleading when interpreting *var *transcription.

## Authors' contributions

UG and LA carried out the panning of parasites, the RT-qPCR and transcription analysis. UG, LA and GW conceived the design of the study, wrote and approved the final manuscript.
